# Implementation of Uterine Artery Doppler Scanning: Improving the Care of Women and Babies High Risk for Fetal Growth Restriction

**DOI:** 10.1155/2023/1506447

**Published:** 2023-01-23

**Authors:** Emmanuel Ekanem, Faris Karouni, Emmanuoil Katsanevakis, Habiba Kapaya

**Affiliations:** ^1^Obstetrics and Gynaecology, United Lincolnshire Hospitals NHS Trust, Lincoln LN2 5QY, UK; ^2^Queen's Medical Centre, Nottingham University Hospitals, Nottingham, UK NG7 2UH; ^3^United Lincolnshire Trust Hospitals, Lincoln County Hospital, Lincoln, UK LN2 5QY

## Abstract

**Introduction:**

While stillbirth rates have declined in many countries, these declines are less marked in the UK. Fetal growth restriction (FGR) affects about 3% to 7% of all pregnancies and is by far the single strongest risk factor for stillbirth. FGR implies a pathological restriction of the genetic growth potential and is not synonymous with small-for-gestational age (SGA). The Royal College of Obstetricians and Gynaecologists (RCOG) defines SGA as an estimated fetal weight (EFW) or abdominal circumference (AC) less than the 10th centile. The likelihood of FGR is higher in severe SGA defined as an EFW or AC less than the 3rd centile. The second version of Saving Babies' Lives Care Bundle (SBLCBv2) recommends the second trimester uterine artery Doppler (UtAD) pulsatility index (PI) screening for pregnancies at high risk of FGR. This study was aimed at determining the prevalence of FGR and assess pregnancy outcomes following the implementation of UtAD at the United Lincolnshire Hospitals NHS Trust (ULHT).

**Methods:**

One-year retrospective cohort study (1st September 2020-31st August 2021) was conducted across both ULHT hospitals in the UK (Lincoln County Hospital in Lincoln and Pilgrim Hospital in Boston).

**Results:**

During the study period, 5197 women were booked at ULHT. Of 5197, 349 were identified as high risk for FGR. When numbers were compared for the two hospitals, FGR rate was higher in Lincoln 8.10% vs. 4.51% in Boston. In addition, an increased proportion of abnormal UtAD scans was observed in Lincoln (35.7%) vs. in Boston (22%) (*P* = 0.014). Of the 349 UtAD scans, 237 were normal (67.9%), 41 showed unilateral notching (11.7%), 43 bilateral notching (12.3%), and 28 raised PI (8%). Babies in the bilateral notching group exhibited the lowest birth weight (*P* = 0.005), born at an earlier gestation (*P* = 0.029), and with low Apgar scores at 1 (*P* = 0.007) and 5 minutes (*P* < 0.001). *Discussion*. UtAD is a useful second trimester screening tool for women identified as high risk for FGR and helps stratify the intensity of surveillance. However, the findings call into question a focus solely on the UtAD PI for improving FGR detection without taking into account bilateral notching.

## 1. Introduction

Fetal growth restriction (FGR—sometimes known as IUGR) is one of the leading causes of stillbirth; hence, detection and monitoring of pregnancies that are high risk for FGR is key in reducing adverse pregnancy outcomes [1–4].

Although the rate of stillbirth in England and Wales has declined from 5.7/1000 births in 2003 to 4.7/1000 in 2013, stillbirth rate in the United Kingdom (UK) remains the highest in developed countries with FGR accounting for half of the stillbirths [5].

Reducing stillbirth is a national priority, and there has been concerted effort to detect and improve the care of women and babies at risk of FGR and stillbirth. In order to achieve this, a number of national initiatives are undertaken; the Saving Babies' Lives Care Bundle being a key [1, 5–7].

Version two of the Saving Babies' Lives Care Bundle (SBLCBv2) has been produced to build on the achievements of version one and is the next step on the journey towards meeting the national ambition to halve stillbirths and neonatal baby deaths by 2025. The updated element 2 of SBLCBv2 focuses more attention on pregnancies at highest risk of FGR, recognises the importance of adequate risk assessment of all women, and has introduced the Uterine Artery Doppler (UtAD) screening tool to help triage women at high risk for placental dysfunction [1, 8].

One of the commonly used indices in UtAD studies is pulsatility index (PI), and this is calculated by subtracting the endiastolic flow from the peak systolic flow and dividing it by the mean [9]. Low endiastolic velocities and early notch are the characteristic waveforms of the uterine artery in nonpregnant women and pregnant women in the first trimester [10–12]. Persistent notching or abnormal velocity ratio is a reflection of increased impedance in the vessel and are associated with inadequate trophoblastic invasion [12]. In unselected pregnant patients, uterine artery notching has been reported to be a better predictor than a resistance index (RI) with predictive values of up to 31% [12–14]. Accuracy in the prediction of FGR is fundamental in the efficient allocation of resources for monitoring and prevention of adverse perinatal outcomes [15, 16]. The SBLCBv2 uses the PI value of UtAD for the surveillance of women at an increased risk of FGR. However, the predictive accuracy of UtAD has been greeted with mixed reactions and its value as a predictive tool has also been questioned [10, 17].

The SBLCBv2 was implemented at the United Lincolnshire Hospital NHS Trust (ULHT) on 1st of September 2020. The aims of our study were to (1) determine the prevalence of FGR and (2) evaluate the outcomes of pregnancies identified as high risk for FGR.

## 2. Materials and Methods

This was a one-year retrospective cohort study conducted at the ULHT across both hospitals' sites (Lincoln County Hospital in Lincoln and Pilgrim Hospital in Boston) of the Department of Obstetrics and Gynaecology.

For the purpose of this study, we have used the definition of severe SGA (an estimated fetal weight (EFW) or abdominal circumference (AC) of less than 3rd centile) from the Royal College of Obstetricians and Gynaecologists (RCOG) to describe FGR [18].

**Figure 1:**
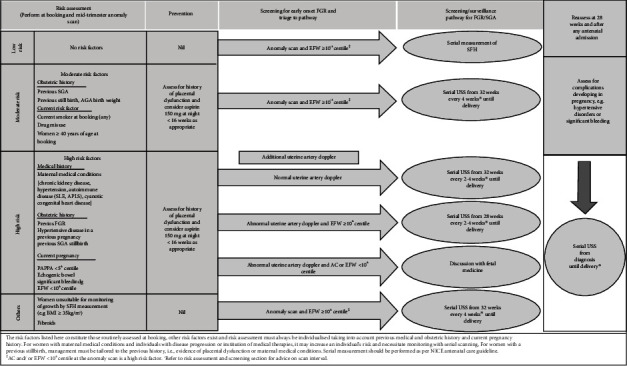
Algorithm describing uterine artery Doppler as a screening tool for risk of early-onset FGR [1].

All women who attended antenatal clinic (ANC) for their dating scan were screened and assessed by the clinic midwives for their risk of placental dysfunction. The risk assessment and surveillance pathway from SBLCBv2 (see Figure 1) was used to stratify women as low, moderate, and high risk of FGR [1].

All pregnancies that were identified as high-risk FGR at the first ANC visit from 1st September 2020 to 31st August 2021 were included in the study.

Based on the recommendations and following the algorithm (Figure 1), all women who were assessed as high risk for FGR were offered UtAD scans from 20 to 24 weeks gestation. The UtAD scans were undertaken by the trained sonographers who used the Gomez et al. reference ranges for the UtAD mean PI for screening patients at risk of placenta-associated diseases and/or FGR [19].

Maternal demographics, BMI, smoking at booking, referral to smoking cessation services, smoking at delivery, UtAD (classified in 4 categories as normal, raised PI, unilateral, and bilateral notching), consideration of aspirin, fetal growth scans, mode of delivery, gestational age at delivery, birth weight, infant's sex, admission to neonatal intensive care unit (NICU), cord gases, and Apgar scores were collected from the maternity electronic database (MEDWAY) and extracted into a prespecified Excel sheet/pro forma.

Statistical analysis was performed using SPSS version 26. Differences between categorical groups were analysed using the chi-square test or Mann–Whitney *U* test for normal and nonnormal distributions of data, respectively. Continuous variables were analysed using the Student *t*-test, Mann–Whitney *U* test or Kruskal-Wallis test, depending on the normality of the data. A *P* value of <0.05 was considered statistically significant.

This study was conducted as a service evaluation project, so formal ethical approval was not required. However, the study was registered with the Clinical Effectiveness Unit (registration: L0402).

## 3. Results

During the study period, 5197 women were booked at ULHT (3183 in Lincoln and 2014 at Pilgrim hospital, Boston). Of 5197, 349 women were identified as high risk for FGR [Lincoln 258 (73.95%) and Boston 91 (26.1%)].

Prevalence of high-risk FGR at ULHT was 6.7%; however, when numbers were compared for the individual sites' prevalence of FGR, it was two times higher in Lincoln 8.10% vs. in Boston 4.51%.

Hundred percent of the pregnancies were correctly risked assessed for FGR across both sites and offered UtAD and third-trimester fetal biometry scans.

Data was compared between Lincoln and Boston sites for a series of demographic and outcome variables (Table 1). Statistical analysis showed that demographics and perinatal outcomes were broadly comparable at both hospitals. However, women booked in Lincoln were older than in Boston (*P* = 0.038) and babies born at Lincoln demonstrated significantly low Apgar scores at 5 minutes (*P* < 0.001) compared to those delivered in Pilgrim hospital. In addition, we found high proportion of abnormal UtAD scans (35.7%) in Lincoln vs. in Boston (22%) (*P* = 0.014).

The two cohorts (Lincoln and Boston) were combined, and the association of UtAD status (normal, unilateral notching, bilateral notching, and raised PI) with a series of outcome variables was tested (Table 2).

Of the 349 UtAD scans performed, 237 were normal (67.9%), 41 showed unilateral notching (11.7%), 43 bilateral notching (12.3%), and 28 had raised PI (8%).

Table 2 demonstrates that babies in the bilateral notching group had the lowest birth weight (*P* = 0.005), were born at an earlier gestation (*P* = 0.029), and had low Apgar scores at 1 (*P* = 0.007) and 5 minutes (*P* < 0.001).

Given that some of the abnormal categories of the UtAD overlapped (for example, some unilateral and bilateral notching had raised PI), we decided to rerun the analysis (Table 3) to assess whether bilateral notching with normal PI was still associated with the adverse outcomes. We excluded all the UtAD with notching (unilateral and bilateral) and raised PI. The overall sample size was reduced for this part of the analysis and included 237 patients with normal UtAD (76.7%), 22 with unilateral notching (7.1%), 22 with bilateral notching (7.1%), and 28 with raised PI (9.1%).

Results from Table 3 indicate that bilateral notching with normal PI did not show statistically significant association with gestational age at delivery, Apgar scores, and birth weight. However, it was interesting to note that babies in the bilateral notching with normal PI exhibited low Apgar scores and were born at an earlier gestation compared to the other UtAD categories.

Sadly, there were two stillbirths in the study cohort, one at each hospital at 26 and 31 weeks' gestation. However, both had normal UtAD.

## 4. Discussion

FGR is a significant cause of perinatal morbidity and mortality, and despite advances in obstetric care, management of FGR remains a major problem in developed countries.

Risk assessment in early pregnancy is important to triage the care of women at increased risk of FGR, and this can be done using UtAD screening in the second trimester between 20 and 24 weeks [20].

Our study demonstrated that all women booked at ULHT were risk assessed for FGR in accordance to the SBLCBv2 and were offered appropriate clinical pathway for FGR surveillance. The commonest indication for classifying women as high risk for FGR was a previous history of FGR, followed by low PAPP-A (pregnancy-associated plasma protein A) and hypertensive diseases in previous pregnancies. Other indications for undertaking UtAD scans are outlined in the supplementary file (available here).

We found a high prevalence of FGR at ULHT (6.7%), particularly at Lincoln (8.1%) compared to the figure reported in literature (3-7%) [21, 22].

For a hospital that serves a small cohort of the UK population, higher rates of FGR indicates an increased clinical workload and the complexity of cases seen in the Trust.

Interestingly, women booked at Lincoln were older and demonstrated abnormal UtAD scans compared to the cohort studied in Boston. This calls to question whether there is an association of maternal age with abnormal UtAD. Studies have demonstrated a discordance in the relationship between maternal age and abnormal UtAD indices. Pirhonen et al. observed abnormal indices of UtAD in women above 35 years of age. However, Papageorghiou et al. and Chanthasenanont found no relationship between abnormal UtAD and maternal age [4, 23, 24].

Growth restricted fetuses are at risk of adverse pregnancy outcomes and early-onset FGR carries at least one of the greatest risks for prematurity or low birth weight [25]. Both early and late FGR have been associated with adverse short- and long-term neurodevelopmental outcomes with cardiovascular and metabolic disorders seen in fetuses with birth weight of <3rd centile and born at <26 weeks gestation [26, 27].

When we compared the various categories of UtAD indices with perinatal outcomes, bilateral uterine artery notching was associated with low birth weight, early gestational age at delivery, and poor Apgar scores. This finding opens a discussion on whether raised PI as per the SBLCBv2 should be classified as abnormal without taking into account the notching factor. The ULHT team agreed to consider notching (unilateral or bilateral) as abnormal and offered fetal biometry scans from 28 weeks whereas scans that reported UtAD as normal had routine scanning from 32 weeks gestation.

There is ample evidence in the literature to suggest the poor outcome associated with notching in the uterine artery [17, 28], and if we had not included notching in the abnormal category, we could have potentially missed on a significant proportion of high-risk babies who required an intervention [25].

Our results corroborate findings from other studies on the value of notching in the uterine artery and the prediction of adverse outcomes. In agreement with our study, Ratiu et al. in their work on uterine artery indices at 19-22 weeks and the predication of pregnancy outcome found that women with the presence of notch and high PI/RI had higher prevalence of growth-restricted babies, low Apgar scores at the 1 and 5 minutes, increased risk of placental insufficiency, placental abruption, low birth weight, and increased need of operative delivery [25]. They also discovered that bilateral notching was more associated with FGR [25]. This was supported by the work carried out by other researchers who assessed the association of UtAD indices and obstetric outcomes [17, 29].

Harrington et al. appraised 1326 ultrasounds in unselected pregnancies who had UtAD between 19 and 24 weeks of gestation. The sensitivity of bilateral notching for predicting pregnancy-induced hypertension and small-for-gestational age (SGA) for patients requiring delivery before 34 weeks was 81.2% and 57.6%, respectively, with positive predictive value of 31.32% for SGA and 37.5% for adverse outcomes [10]. The authors also noted that pregnancies with notching had a 1 : 2 chance of developing adverse outcomes and a 1 : 3 chance of developing complications necessitating delivery before 34 weeks. When the groups were subdivided into unilateral and bilateral notching, the sensitivity of unilateral notching in predicting SGA was poor compared to that of bilateral notching. There was a high sensitivity of bilateral notching in predicting early delivery, and this was also seen in our study [10].

Secondary finding that emerged from the study was the observation of high prevalence of smoking in pregnant women and poor uptake of the stop-smoking services at ULHT.

All women who smoked at booking were referred to the stop-smoking service. However, only 26.7% accepted and engaged with the smoking cessation services. When we looked at the smoking status at delivery, of the 101 women who were booked as smokers, 80 (79.2%) continued to smoke throughout pregnancy and delivery.

This data is shocking and raises a question on the efficacy and sensitivity of the smoking cessation service and possibly explain high prevalence of FGR in our population compared to the national stats. In addition, when we investigated the two stillbirths, we found that both were offered UtAD and fetal biometry scans in accordance to the SBLCBv2. However, both patients continued to smoke in pregnancy and presented with placental abruption and preterm labour at 26 and 31 weeks gestation leading to a devastating outcome (stillbirth). Findings from the study urge to deliver a different service for tobacco dependency treatment for pregnant smokers at ULHT, in line with NICE [21, 22, 30].

On a more positive note, ULHT was the first in the midland region to implement UtAD as a screening tool for triaging care for women identified as high risk for FGR. Our biggest challenge prior to implementing SBLCBv2 was the capacity to undertake the UtAD scans. The care pathway was launched at ULHT when maternity services were impacted by the COVID-19 pandemic. The speed at which change was effected was a reflection of continuous support, education, and strong leadership that inspired colleagues to ensure that our services offer the best possible care and compassion to our women and their families.

The electronic MEDWAY enabled us to pull an automatic report for number of variables studied instead of manual extraction of the data, minimising error and bias in reporting the outcomes.

The main limitation of the study was its retrospective design, the inability to control the data quality, and potential inconsistency in record-keeping across time and clinical staff. However, this meant that data were entered without the knowledge of the project, therefore reducing the risk of bias.

In summary, the study supports the implementation of UtAD for predicting high-risk FGR pregnancies destined for adverse outcome and enabled to stratify the intensity of fetal surveillance. However, the debate over whether to use uterine artery PI alone or in conjunction with notching continues and more research are required to support or refute the use of uterine artery notching in screening women at high risk for FGR. Nonetheless, based on our findings, we advise uterine artery notching to be reported in clinical practice for appropriate counselling and managing pregnancies at high risk of FGR.

## Figures and Tables

**Table 1:** Effect of hospital location on demographic and outcome variables.

Variable	Lincoln	Boston	*P* value
Age	31.0 (5.27)	30.0 (5.31)	0.038
Parity (% multiparous)	219 (84.9)	72 (79.1)	0.27
BMI	28.9 (6.81)	28.2 (7.97)	0.08
UtAD (% normal)	166 (64.3)	71(78)	0.014
Gestational age at delivery (weeks)	38.1 (1.81)	38.4 (1.39)	0.39
Aspirin (% positive)	248 (96.1)	84 (92.3)	0.22
Induction (% induced)	108 (41.9)	33 (36.3)	0.42
Mode of delivery (%vaginal)	159 (61.6)	59 (64.8)	0.65
Birth weight (kg)	3.13 (0.59)	3.1 (0.52)	0.41
Apgar 1 min	8.59 (1.34)	8.70 (1.29)	0.12
Apgar 5 min	8.97 (0.94)	9.59 (0.52)	<0.001
NNU (% positive)	46 (18)	16 (17.8)	0.97
Smoker at booking (%)	70 (27.1)	31 (34.1)	0.21
Smoking referral offered (%)	258 (100)	91 (100)	1
Smoking referral accepted (% of smokers)	17 (25.4)	10 (33.3)	0.42
Still smoker at delivery (% of smokers)	58 (86.6)	22 (73.3)	0.12

**Table 2:** Association of normal and abnormal uterine artery Doppler with perinatal outcomes.

Variable	Normal	Unilateral notching	Bilateral notching	Raised PI	*P* value
Mode of delivery (% vaginal)	190 (80.2)	32 (78)	35 (81.4)	20 (71.4)	0.74
Induction (% induced)	110 (46.4)	19 (46.3)	23 (53.5)	21 (75)	0.14
Birth weight (kg)	3.17 (0.55)	3.26 (0.52)	2.83 (0.68)	3.00 (0.56)	0.005
Gestational age at delivery (weeks)	38.4 (1.49)	38.4 (1.53)	37.3 (2.72)	38.1 (1.41)	0.029
Apgar 1 min	8.62 (1.32)	8.97 (0.28)	8.13 (1.87)	8.79 (0.63)	0.007
Apgar 5 min	9.06 (0.85)	9.15 (0.37)	8.85 (1.53)	9.58 (0.51)	0.001

**Table 3:** Association of normal, unilateral notching, bilateral notching, and normal PI uterine artery Doppler with perinatal outcomes.

Variable	Normal	Unilateral notching	Bilateral notching	Raised PI	*P* value
Mode of delivery (% vaginal)	140 (63.3)	14 (63.6)	10 (45.5)	20 (71.4)	0.64
Induction (% induced)	86 (38.9)	9 (40.9)	12 (54.5)	9 (32.1)	0.44
Birth weight (kg)	3.15 (0.53)	3.02 (0.68)	3.16 (0.67)	3.26 (0.64)	0.49
Gestational age at delivery (weeks)	38.3 (1.50)	38.2 (1.61)	37.9 (2.76)	38.4 (1.20)	0.74
Apgar 1 min	8.63 (1.31)	8.67 (1.06)	8.55 (1.40)	8.77 (0.91)	0.65
Apgar 5 min	9.14 (0.79)	9.05 (0.59)	8.91 (0.68)	9.23 (0.65)	0.52

## Data Availability

The datasets used and/or analysed are available from the corresponding author on reasonable request.

## Supplementary Materials

Supplementary Materials Supplementary Table: indications for UtAD scans performed at ULHT during the study (1st September 2020-31st August 2021).
