# Supplementary material for: Implementation of Uterine Artery Doppler Scanning: Improving the Care of Women and Babies High Risk for Fetal Growth Restriction
Source: J Pregnancy. 2023 Jan 23;2023:1506447. doi: 10.1155/2023/1506447 (PMC9886456; doi:10.1155/2023/1506447)
Supplement: Supplementary Materials — Supplementary Table: indications for UtAD scans performed at ULHT during the study (1st September 2020-31st August 2021). [file 1506447.f1.docx]

**Supplementary file**

**Supplementary table: Indications for UtAD scans performed at ULHT during the study (1^st^ September 2020-31^st^ August 2021)**

| **Indications for UtAD** | **Number of scans in Lincoln (n=258)** | **Number of scans in Boston (n=91)** | **Total number at ULHT (n=349)** |
| --- | --- | --- | --- |
| Previous FGR | 103 | 40 | 143 |
| Low Papp-A | 48 | 21 | 69 |
| Hypertensive disease in previous pregnancies | 51 | 14 | 65 |
| Previous SGA with history of stillbirth | 11 | 6 | 17 |
| Multiple risk factors (previous SGA + smoker, hypertension/type 2 diabetes | 30 | 5 | 35 |
| Essential hypertension | 8 | 4 | 12 |
| Echogenic bowel | 2 | 1 | 3 |
| Chronic kidney disease | 2 |  | 2 |
| Raised intracranial hypertension | 1 |  | 1 |
| Autoimmune disease | 1 |  | 1 |
| Congenital heart disease | 1 |  | 1 |
